# Wand-Based Calibration of Unsynchronized Multiple Cameras for 3D Localization

**DOI:** 10.3390/s24010284

**Published:** 2024-01-03

**Authors:** Sujie Zhang, Qiang Fu

**Affiliations:** 1 Tianjin College, University of Science and Technology Beijing, Tianjin 301830, China; zsj6618@126.com; 2 School of Intelligence Science and Technology, University of Science and Technology Beijing, Beijing 100083, China; 3 Institute of Artificial Intelligence, University of Science and Technology Beijing, Beijing 100083, China; 4 Key Laboratory of Intelligent Bionic Unmanned Systems, Ministry of Education, University of Science and Technology Beijing, Beijing 100083, China

**Keywords:** camera calibration, unsynchronized multi-camera system, timestamp, 3D localization

## Abstract

Three-dimensional (3D) localization plays an important role in visual sensor networks. However, the frame rate and flexibility of the existing vision-based localization systems are limited by using synchronized multiple cameras. For such a purpose, this paper focuses on developing an indoor 3D localization system based on unsynchronized multiple cameras. First of all, we propose a calibration method for unsynchronized perspective/fish-eye cameras based on timestamp matching and pixel fitting by using a wand under general motions. With the multi-camera calibration result, we then designed a localization method for the unsynchronized multi-camera system based on the extended Kalman filter (EKF). Finally, extensive experiments were conducted to demonstrate the effectiveness of the established 3D localization system. The obtained results provided valuable insights into the camera calibration and 3D localization of unsynchronized multiple cameras in visual sensor networks.

## 1. Introduction

Currently, multi-camera localization is used in many fields, e.g., the currently hot field of autonomous driving [1,2]. But, in many real-world scenarios such as swarm formations and mobile robots, multiple cameras often constitute an unsynchronized multi-camera localization system (UMCLS) without additional synchronization processing [3]. The main challenge posed by unsynchronized cameras is that each scene point would be captured at different instants by each camera. This would induce triangulation errors because the two image rays would either intersect at an incorrect location or simply not intersect at all [4]. Therefore, unsynchronization would bring large errors to traditional multi-camera calibration and localization algorithms, which are designed on the basis of synchronized cameras. This paper addresses the challenge posed by unsynchronized cameras by using timestamp matching and pixel fitting.

For a UMCLS, the first step is to perform multi-camera calibration, which aims to accurately compute the intrinsic parameters (principal point, lens distortion, etc.) and the extrinsic parameters (rotation matrix and translation vector between the camera coordinate system and the reference coordinate system) of each camera. Multi-camera calibration is the basis of 3D localization since the calibration results would be subsequently used during the process of 3D localization. The localization accuracy of a UMCLS will be determined by the calibration accuracy of multiple cameras directly, so the process of multi-camera calibration is very important. According to the dimension of the calibration object, existing multi-camera calibration methods can be roughly divided into five kinds: methods based on 3D calibration objects [5,6], methods based on 2D calibration objects [7,8], methods based on 1D calibration objects [9], methods based on point objects [10], and self-calibration methods [11,12,13,14,15,16]. Note that methods based on 1D calibration objects can quickly and easily complete the calibration of multiple cameras without being affected by occlusion [17], so this paper chooses to use the 1D calibration method.

Until now, most of the multi-camera calibration methods are developed for synchronized cameras, and synchronization is controlled by a hardware trigger. But, in many cases, the camera frame rates are different, the cameras work asynchronously, and the acquired image sequences are naturally not matched. Therefore, image synchronization and camera calibration are usually carried out simultaneously for multiple unsynchronized cameras. From the perspective of the calibration object, existing calibration methods for unsynchronized cameras could be generally classified into two kinds: methods based on point objects [18,19,20] and methods based on 3D calibration objects [21]. As mentioned above, 1D calibration methods are quite suitable for calibrating multiple cameras, and a practical 1D calibration method needs to be designed for a UMCLS.

On the other hand, 3D localization is a crucial function for unsynchronized multiple cameras, and there are some related research works. For example, Benrhaiem et al. [22] proposed a temporal offset-invariant 3D reconstruction method to solve the problem of camera unsynchronization. Their approach only deals with stereo cameras based on the epipolar geometry. Piao and Sato [23] proposed a method to achieve the synchronization of multiple cameras and compute the epipolar geometry from uncalibrated and unsynchronized cameras. In particular, they used the affine invariance on the frame numbers of camera images to find the synchronization. Considering that 3D localization needs to meet the real-time requirement in practice, the acquired unsynchronized multi-camera images should be quickly processed. Therefore, a fast and high-precision feature point localization method needs to be designed for a UMCLS.

In this paper, in order to solve the problem of unsynchronization, we propose a time synchronization scheme and a wand-based calibration method to complete the calibration of multiple perspective/fish-eye cameras. Then, we propose an EKF-based unsynchronized 3D localization method. Finally, we built a real UMCLS and verified its performance by using a flapping-wing micro air vehicle (FWAV) to accomplish fixed-height experiments. The main contributions of this study are as follows:

(1) For unsynchronized perspective or fish-eye cameras, a wand-based multi-camera calibration method is proposed by using timestamp matching and pixel fitting, and the calibration result was verified by real experiments.

(2) An EKF-based 3D localization algorithm is proposed for unsynchronized perspective or fish-eye cameras.

(3) An actual UMCLS was built, and the performance of the system was evaluated through the feature point reconstruction experiments and fixed-height experiments of an FWAV.

The remainder of this paper is organized as follows. The problem formulation, designed calibration algorithm, and designed 3D localization algorithm are introduced in Section 2. Section 3 presents the results and a discussion of the calibration and fixed-height experiments. Section 4 is the conclusion of this paper.

## 2. Materials and Methods

### 2.1. Preliminaries and Problem Formulation

#### 2.1.1. General Camera Model

**Figure 1:**
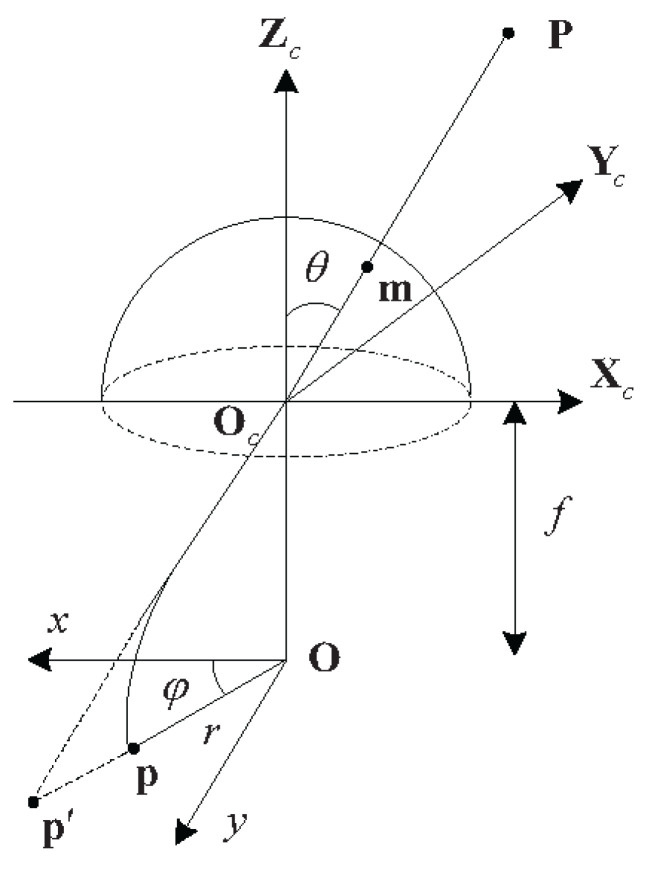
Fish-eye camera model.

In order to broaden the applicability of the proposed methods to different cameras, this paper adopts the general camera model proposed by Kannala et al. [24], which is suitable for perspective cameras, as well as fish-eye cameras. As shown in Figure 1, the real fish-eye lense does not completely follow the conventional perspective model. The following is a general form of prediction for imaging:

(1) $$r\left(\theta \right)=k_{1}\theta +k_{2}\theta^{3}+k_{3}\theta^{5}+k_{4}\theta^{7}+k_{5}\theta^{9}+\ldots$$

where θ is the angle between the principal axis and the incident light, *r* is the distance between the image point and the principal point, even powers are removed in order to extend *r* to the negative semi-axis as an odd function, and odd powers span the set of continuous odd functions. Note that the first five items already have enough degrees of freedom to approximate different projection curves. Therefore, the radially symmetric part of the camera model only contains the first five parameters $k_{1},k_{2},k_{3},k_{4},k_{5}$.

Let *F* be the mapping from the incident light to standardized image coordinates:

(2) $$\left[\begin{array}{c} x\\ y \end{array}\right]=r\left(\theta \right)\left[\begin{array}{c} \cos\varphi \\ \sin\varphi \end{array}\right]=F\left(\Phi \right)$$

where r(θ) contains the projection model of the first five items of Equation (1) and Φ=(θ,φ) is the direction of the incident light. For a real lens, the value of the parameter $k_{i}$ makes r(θ) monotonically increase over the interval $\left[0,\theta_{\max}\right]$, where $\theta_{\max}$ is the maximum angle of view. Therefore, when calculating the inverse of *F*, the roots of the ninth-order polynomial can be found numerically and, then, real roots between 0 and $\theta_{\max}$ can be selected.

Assuming that the pixel coordinate system is orthogonal, we can obtain the pixel coordinate ${\left[u,v\right]}^{T}$:

(3) $$\left[\begin{array}{c} u\\ v \end{array}\right]=\left[\begin{array}{cc} m_{u} & 0\\ 0 & m_{v} \end{array}\right]\left[\begin{array}{c} x\\ y \end{array}\right]+\left[\begin{array}{c} u_{0}\\ v_{0} \end{array}\right]$$

where ${\left[u_{0},v_{0}\right]}^{T}$ is the center point, that is the pixel coordinate corresponding to the center of the imaging plane, and $m_{u}$ and $m_{v}$ are the number of pixels per unit distance in the horizontal and vertical directions, respectively.

By combining (2) and (3), we obtain the forward camera model:

(4) $$m=P_{c}\left(\Phi \right)$$

where $m={\left[u,v\right]}^{T}$. The P9$\left(k_{1},k_{2},k_{3},k_{4},k_{5},u_{0},v_{0},m_{u},m_{v}\right)$ camera model is used in this paper. The nine parameters consist of the five parameters of the radially symmetrical part, the center point, and the number of pixels per unit distance in the horizontal and vertical directions.

#### 2.1.2. Problem Formulation

**Figure 2:**
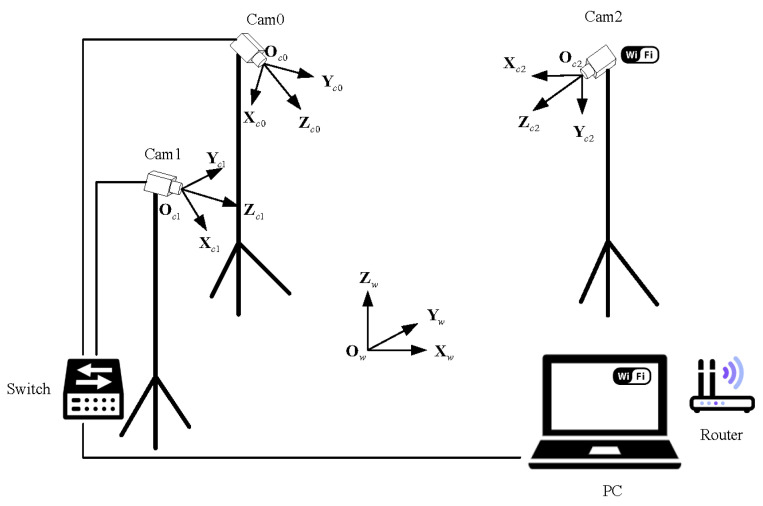
System schematic.

**Figure 3:**
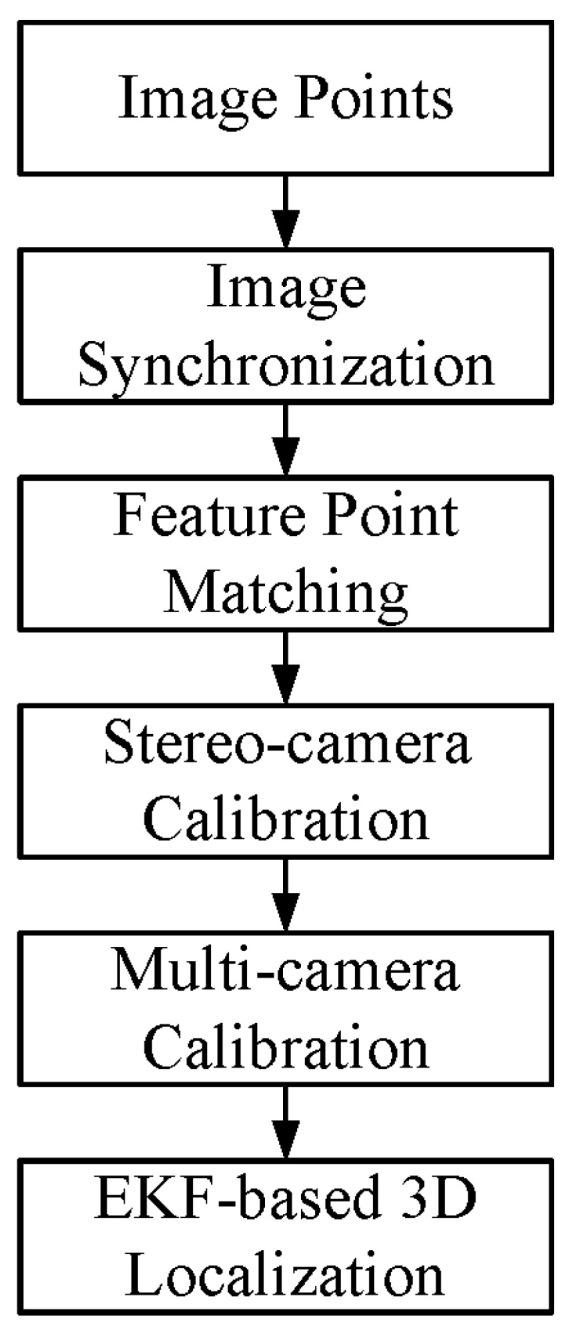
Flowchart of the proposed camera calibration and 3D localization methods.

As shown in Figure 2, suppose we use two wired cameras and one wireless camera in our unsynchronized multi-camera system, and their camera coordinate systems are $O_{c_{i}}-X_{c_{i}}Y_{c_{i}}Z_{c_{i}}\left(i=0,1,2\right)$. The world coordinate system $O_{w}-X_{w}Y_{w}Z_{w}$ is determined manually (generally, on the ground). In this paper, our main work was to first calibrate the internal and external parameters of the unsynchronized cameras and obtain the conversion relationship between the camera coordinate system $O_{c_{i}}-X_{c_{i}}Y_{c_{i}}Z_{c_{i}}$ and the world coordinate system $O_{w}-X_{w}Y_{w}Z_{w}$. Then, complete the 3D localization of the feature points by using the unsynchronized cameras and accomplish real-time tracking of the feature points. Finally, a UMCLS was built to complete the fixed-height control of an FWAV to verify the system performance. The flowchart of the proposed multi-calibration method and the EKF-based 3D localization algorithm is shown in Figure 3.

### 2.2. Calibration Algorithm

This section is the core of the methods to solve the unsynchronized problem. The idea is that the acquired camera images are pre-processed or synchronized so that the unsynchronized multi-camera calibration based on the 1D wand could be completed. In the following, the two-camera and multi-camera situations are separately processed for image synchronization, and the corresponding processing methods are designed for the loss of marker points.

#### 2.2.1. Pre-Processing for Two Cameras

**Figure 4:**
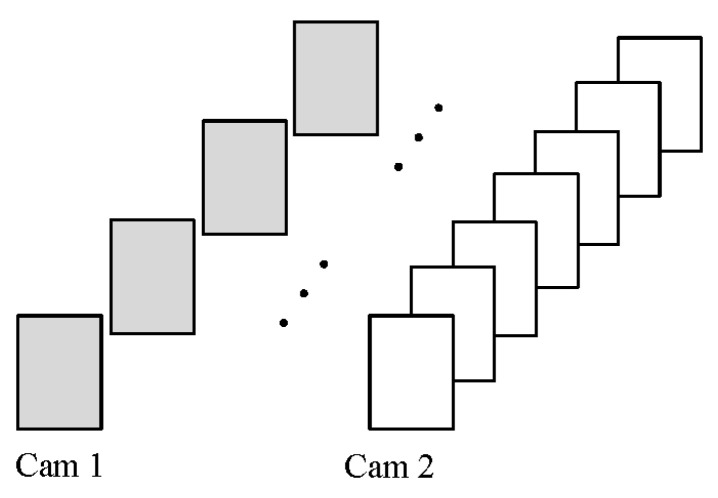
Unsynchronized camera image sequences.

**Figure 5:**
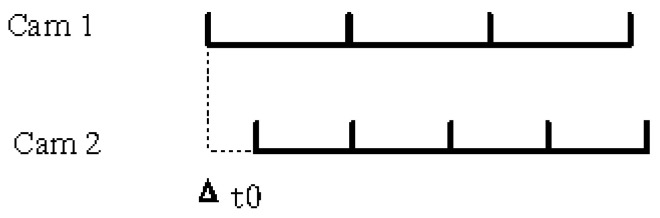
Timestamp line segment.

**Figure 6:**
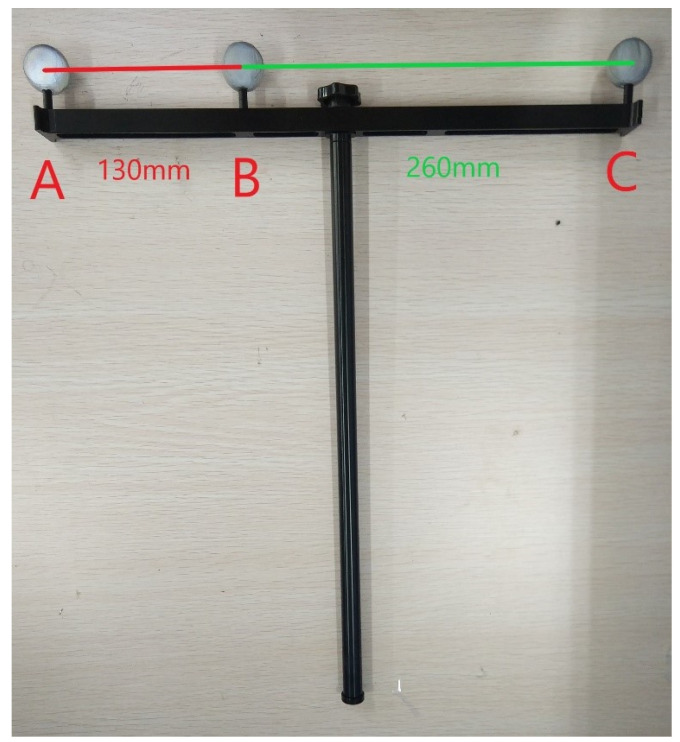
The 1D calibration wand.

As shown in Figure 4, since the frame rates of the two cameras are different, we can obtain the image sequences of the two cameras. Suppose that Cam1 with a low-frame-rate image sequence is used as the benchmark; we hope to obtain the image frames matching the image sequence of Cam1 from the image sequence of Cam2. Assume that the frame rates of Cam1 and Cam2 are M fps and N fps, respectively. As shown in Figure 5, the two line segments represent the shooting time sequence of the two cameras, and the end points on the line segment represent the time when the camera captures the images. Δt0 represents the time delay between the first frame of the two acquired images. Since the image sequence of Cam1 with a low frame rate is selected as the reference, the image sequence information of Cam1 is completely retained:

(5) Cam1_new[i]=Cam1[i]

where *i* is the serial number of the image, denoting that it is the *i*th image obtained by Cam1. Cam1[i] represents the coordinate information of all the marker points of the *i*th image frame. Since the 1D wand used in this paper (see Figure 6) has three markers, Cam1[i] denotes the pixel coordinates of the three markers. Cam1_new[i] represents the new image information of the *i*th image frame after pre-processing.

Assuming that the frame rates of the two cameras remain unchanged, the following formula can be obtained:

(6) $$\Delta t=\left(\frac{i}{M}+\Delta t0\right)\times \;N-\left\lfloor \left(\frac{i}{M}+\Delta t0\right)\times \;N\right\rfloor$$

where ⌊⌋ means rounding down, that is obtaining the largest integer no greater than the number in it, and Δt represents the ratio of the time of the two image frames in Cam2 corresponding to the *i*th image frame of Cam1. Then, we use the interpolation fitting method to obtain:

(7) $$\begin{array}{c} \;\mathrm{Cam}2\_\mathrm{new}\left[i\right]=\left(1-\Delta t\right)\times \mathrm{Cam}2\left[\lfloor \left(\frac{i}{M}+\Delta t0\right)\times N\rfloor \right]\\ +\Delta t\times \mathrm{Cam}2\left[\lfloor \left(\frac{i}{M}+\Delta t0\right)\times N\rfloor +1\right] \end{array}$$

where *i* is the serial number of the image and Cam2_new[i] represents the new image information of the *i*th image frame after processing. Note that the above equations are derived under ideal conditions. It is necessary to ensure that the frame rate of each camera is stable and the initial Δt0 can be accurately measured. However, these two conditions are difficult to guarantee in practice and rely too much on the accuracy of the initial parameters. Once a certain value has errors or changes, it will have a great impact on the subsequent calculations so that the synchronization effect cannot be achieved. So, we simplified the method by stamping every frame of the images from each camera with a timestamp.

(8) $$\mathrm{Cam}2\_\mathrm{new}\left[N_{t}\right]=\frac{\left(t_{2}-t\right)\times \mathrm{Cam}2\left[N\right]+\left(t-t_{1}\right)\times \;\mathrm{Cam}2\left[N+1\right]}{\left(t_{2}-t_{1}\right)}$$

As shown in Equation (8), t is the timestamp corresponding to the $N_{t}$th frame in Cam1. Search the images captured by Cam2 for two adjacent frames with timestamps satisfying $t_{1}<t\leq t_{2}$, where $t_{1}$ and $t_{2}$ correspond to the timestamps of the Nth image frame Cam2[N] and the (N+1)th image frame Cam2[N+1], respectively.

Although each image frame needs to be timestamped, the time error will not accumulate. In addition, since the timestamp is added when receiving an image on the ground station, there is no doubt that the built-in clocks of the cameras are unsynchronized. However, the time delay of transmitting image data is unstable for each camera, and this problem will be solved in the subsequent optimization of the camera parameters.

#### 2.2.2. Pre-Processing for Multiple Cameras

Referring to the synchronization method of two cameras, select the camera with the lowest frame rate as the reference for multiple cameras, so as to obtain the new image sequences matching the image sequences of the other cameras and the reference camera.

(9) $$\begin{array}{c} \mathrm{Camn}\_\mathrm{new}\left[N_{t}\right]=\frac{\left(t_{n2}-t\right)\times \mathrm{Cam}n\left[N_{n}\right]+\left(t-t_{n1}\right)\times \;\mathrm{Cam}n\left[N_{n}+1\right]}{\left(t_{n2}-t_{n1}\right)} \end{array}$$

(10) $$\begin{array}{c} \mathrm{Cam}2\_\mathrm{new}\left[N_{t}\right]=\frac{\left(t_{2}-t\right)\times \mathrm{Cam}2\left[N_{t_{1}}\right]+\left(t-t_{1}\right)\times \;\mathrm{Cam}2\left[N_{t_{2}}\right]}{\left(t_{2}-t_{1}\right)} \end{array}$$

As shown in Equation (9), t is the timestamp corresponding to the $N_{t}$th image frame of the reference camera. Search the images captured by the other cameras for two adjacent frames with timestamps satisfying $t_{n1}<t\leq t_{n2}$, where $t_{n1}$ and $t_{n2}$ correspond to the *n*th camera’s Nth frame image information $\mathrm{Cam}n[N_{n}]$ and (N+1)th frame image information $\mathrm{Cam}n[N_{n}+1]$, respectively.

#### 2.2.3. Loss of Marker

When the marker is occluded or exceeds the camera’s field of view, the camera cannot capture the information of the marker. Only when the three markers are captured by the same camera will we record the marker coordinates; otherwise, this image frame will be regarded as an invalid frame during the calibration process.

In Equation (8), for the timestamp t of the reference camera sequence, if condition $t_{1}<t\leq t_{2}$ is met, it is possible to find that both adjacent frames are not valid frames. So, we need to look for valid frames before $t_{1}$ or $t_{2}$. The formula in the case of invalid frames can be obtained as shown in Equation (10), where $\mathrm{Cam}2[N_{t_{1}}]$ represents the image frame of Cam2 corresponding to the valid timestamp $t_{1}$ and $N_{t_{1}}$ represents the sequence number of the original image sequence of Cam2 at $t_{1}$. $\mathrm{Cam}2[N_{t_{2}}]$ means the same.

This is carried out to obtain as much image data as possible for camera calibration. However, if the matching timestamps are too different, the fitted result will have large errors, so set the rule:

(11) $$\begin{array}{c} \left|t_{1}-t\right|\leq 2/M\\ \left|t_{2}-t\right|\leq 2/M \end{array}$$

where *M* is the frame rate of the current camera.

#### 2.2.4. Multi-Camera Calibration Method

After the above pre-processing is performed on the image sequences acquired by the unsynchronized multi-camera system, the 1D-wand-based multi-camera calibration method proposed in our previous work [25] can be used.

First, the initialization of the camera’s internal parameters needs to be completed. By taking pictures of the calibration board for each camera, the method in [24] can be used to complete the calibration of the internal parameters and obtain the initial internal parameter $\left(k_{1}^{i},k_{2}^{i},m_{u}^{i},m_{v}^{i},u_{0}^{i},v_{0}^{i}\right)$ of each camera.

Then, stereo camera calibration is performed. Given five or more corresponding feature points, the essential matrix can be calculated using the 5-point random sample consensus (RANSAC) algorithm [26]. So, the initial value of the camera’s external parameters ($R_{01},{\bar{T}}_{01}$) can be obtained through singular-value decomposition [27]. It should be noted that ${\bar{T}}_{01}$ is the normalized translation vector, and the real translation vector needs to be obtained further. Then, the internal and external parameters of the cameras are nonlinearly optimized. Set $A_{j}^{r},B_{j}^{r},C_{j}^{r}$ to be the reconstructed space coordinates of A,B,C in the *j*th frame image, respectively. Since the three points A,B,C are on the calibration wand, the error function can be obtained:

(12) $$\begin{array}{cc} \; & g_{1,j}\left(x\right)=L_{1}-∥A_{j}^{r}-B_{j}^{r}∥\\ \; & g_{2,j}\left(x\right)=L_{2}-∥B_{j}^{r}-C_{j}^{r}∥\\ \; & g_{3,j}\left(x\right)=L-∥A_{j}^{r}-C_{j}^{r}∥ \end{array}$$

where $x=(k_{1}^{0},k_{2}^{0},m_{u}^{0},m_{v}^{0},u_{0}^{0},v_{0}^{0},k_{1}^{1},k_{2}^{1},m_{u}^{1},m_{v}^{1},u_{0}^{1},v_{0}^{1},r_{1},$$r_{2},r_{3},t_{x},t_{y},t_{z}{)}^{T}\in R^{18}$. Note that $(r_{1},$$r_{2},r_{3}{)}^{T}\in R^{3}$ is another form of the rotation matrix transformed by the Rodrigues formula [27] and ${\left(t_{x},t_{y},t_{z}\right)}^{T}\in R^{3}$ is the translation vector.

Based on Equation (12), we can obtain the final objective function:

(13) $$x^{*}=\arg\min_{x}\sum_{j=1}^{N}\left(g_{1,j}^{2}\left(x\right)+g_{2,j}^{2}\left(x\right)+g_{3,j}^{2}\left(x\right)\right)$$

which can be solved by using the Levenberg–Marquardt method [27].

The above solution can be refined by bundle adjustment, which involves both camera parameters and 3D space points. Using the bundle adjustment method not only optimizes the measurement error, but also optimizes the problems that cannot be solved during time synchronization. For example, when waving the calibration wand, it cannot be guaranteed to be a perfect uniform linear motion and the timestamps of the acquired images will be affected by the fluctuation of the transmission delay. The above problem can be well solved by bundle adjustment.

Since the 3D space points $A_{j},B_{j}$ and $C_{j}$ are collinear, they have the relation as follows:

(14) $$\left\{\begin{array}{c} B_{j}=f_{B}\left(A_{j},\phi_{j},\theta_{j}\right)=A_{j}+L_{1}\cdot n_{j}\\ C_{j}=f_{C}\left(A_{j},\phi_{j},\theta_{j}\right)=A_{j}+L\cdot n_{j} \end{array}\right.$$

where $L_{1}$ represents the actual length of AB and $n_{j}={\left(sin\phi_{j}cos\theta_{j},sin\phi_{j}sin\theta_{j},cos\phi \right)}^{T}$ denotes the unit vector of the calibration wand. In order to improve the accuracy of the camera model, three more parameters $\left(k_{1},k_{2},k_{3}\right)$ are added to each camera, and their initial values are set to zero. So, the final camera parameters involved in the optimization are:

(15) $$\begin{array}{c} x^{\prime }=\left(k_{1}^{0},k_{2}^{0},m_{u}^{0},m_{v}^{0},u_{0}^{0},v_{0}^{0},k_{3}^{0},k_{4}^{0},k_{5}^{0},k_{1}^{1},k_{2}^{1},m_{u}^{1},\right.\\ {\left.m_{v}^{1},u_{0}^{1},v_{0}^{1},k_{3}^{1},k_{4}^{1},k_{5}^{1},r_{1},r_{2},r_{3},t_{x},t_{y},t_{z}\right)}^{T}\in R^{24} \end{array}$$

Let the function $P_{i}\left(x^{\prime },M\right)$ (*i* = 0, 1) denote that the 3D point M is projected onto the *i*th camera image plane under the parameters $x^{\prime }$. The final optimization problem as shown in Equation (16) is solved by using the sparse Levenberg–Marquardt method [28]. Based on the stereo calibration results, the internal and external parameters of the multi-camera system could be obtained when the number of cameras is more than two (see [25] for details).

(16) $$\begin{array}{c} \min_{x^{\prime },A_{j},\phi_{j},\theta_{j}}\sum_{i=0}^{1}\sum_{j=1}^{N_{1}}\left({∥a_{ij}-P_{i}\left(x^{\prime },A_{j}\right)∥}^{2}+{∥b_{ij}-P_{i}\left(x^{\prime },f_{B}\left(A_{j},\phi_{j},\theta_{j}\right)\right)∥}^{2}+\right.\\ \left.{∥c_{ij}-P_{i}\left(x^{\prime },f_{C}\left(A_{j},\phi_{j},\theta_{j}\right)\right)∥}^{2}\right) \end{array}$$

### 2.3. Three-Dimensional Localization Algorithm

In this section, we propose a real-time tracking algorithm for each feature point based on the extended Kalman filter, which is often used for information fusion of multi-sensor fusion, e.g., the IMU and cameras. The state model adopts the traditional linear model:

(17) $$x_{k}=Ax_{k-1}+\gamma_{k}$$

where $A\in R^{6\times 6}$ represents a block diagonal matrix with each block $\left[1,T_{s};0,1\right]$ ($T_{s}$ denotes the sampling time) and $\gamma_{k}={\left[0,\gamma_{k}^{1},0,\gamma_{k}^{2},0,\gamma_{k}^{3}\right]}^{T}\in R^{6}$ models the motion uncertainties. The measurement model for multiple cameras adopts the model presented in our previous study [29]:

(18) $$z_{k}=g\left(x_{k},\alpha_{i},R_{w}^{c_{i}},T_{w}^{c_{i}}\right)+v_{k}$$

where $z_{k}={\left[z_{k}^{c_{1}}\ldots z_{k}^{c_{N}}\right]}_{k}^{T}\in R^{2N}$ (*N* is the number of cameras), $g\left(\cdot \right)={\left[g^{c_{1}}\left(\cdot \right)\ldots g^{c_{N}}\left(\cdot \right)\right]}_{k}^{T}\in R^{2N}$, $x_{k}=\left[X_{k},V_{x,k},Y_{k},V_{y,k},Z_{k},V_{z,k}\right]\in R^{6}$, $\alpha_{i}\in R^{9}$ is the internal parameters of the *i*th camera, and $v_{k}={\left[\begin{array}{ccc} v_{k}^{c_{1}} & \ldots & v_{k}^{c_{N}} \end{array}\right]}_{k}^{T}\in R^{2N}$ and $\left(R_{w}^{c_{i}},T_{w}^{c_{i}}\right)$ represent the rotation matrix and translation vector from the *i*th camera coordinate system to the world coordinate system, respectively. Note that $\alpha_{i}$ and $\left(R_{w}^{c_{i}},T_{w}^{c_{i}}\right)$ could be obtained by using the calibration algorithm in Section 2.2.

Since the frame rate of each camera is different, it was set as ${\mathrm{FR}}_{i}$. For an actual unsynchronized multi-camera system, this paper selects the reciprocal of the maximum frame rate as the sampling time, that is

(19) $$T_{s}=\frac{1}{max({\mathrm{FR}}_{1},{\mathrm{FR}}_{2},\ldots ,{\mathrm{FR}}_{N})}$$

The sampling time selected in this way can meet the requirements of actual filtering and also reduce the computational burden. In this paper, the prediction equation of the EKF is:

(20) $$\begin{array}{c} {\hat{x}}_{k,k-1}=A{\hat{x}}_{k-1,k-1}\\ P_{k,k-1}=AP_{k-1,k-1}A^{T}+Q_{k-1} \end{array}$$

The correction equation of the EKF is:

(21) $$\begin{array}{cc} H_{k} & ={\left.\frac{\partial G(x)}{\partial x}\right|}_{x={\hat{x}}_{k,k-1}}\\ K_{k} & =P_{k,k-1}H_{k}^{T}{\left(R_{k}+H_{k}P_{k,k-1}H_{k}^{T}\right)}^{-1}\\ {\hat{x}}_{k,k} & ={\hat{x}}_{k,k-1}+K_{k}\left(Z_{k}-G\left({\hat{x}}_{k,k-1}\right)\right)\\ P_{k,k} & =P_{k,k-1}-K_{k}H_{k}P_{k,k-1} \end{array}$$

where $P_{k,k-1}$ is the prior variance of the estimated error, $P_{k,k}$ is the posterior variance of the estimated error, and $K_{k}$ is the Kalman gain matrix at step *k*.

## 3. Results and Discussion

### 3.1. System Construction and Experimental Design

**Figure 7:**
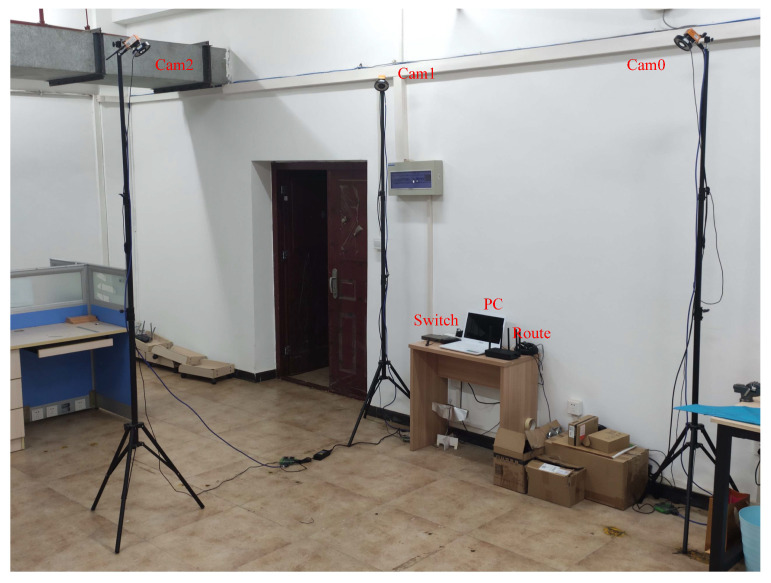
System construction.

As shown in Figure 7, the UMCLS has three cameras. Cam0 and Cam1 communicate with the ground computer through a switch. Cam2 communicates with the ground computer through a router. The two wires connected to Cam2 are used to supply power to the camera and the infrared light source around it, respectively. The frame rates of Cam0 and Cam1 can be adjusted, and the frame rate of Cam2 is constantly set to 110 Hz. In this way, the UMCLS can be used to study the influence of asynchronous connection caused by wired connection and wireless connection (WiFi) and the influence of different camera frame rates.

Note that all the cameras used in this paper are smart cameras, which can pre-process the acquired image and extract the center coordinate of each feature point, so the output of each camera is the center coordinate information of the feature points that have been processed. The proposed methods in this paper are suitable for more than three cameras, and we just took three cameras as an example. The proposed methods are also applicable to outdoor scenarios, but feature detection will be challenging in complex outdoor environments.

**Figure 8:**
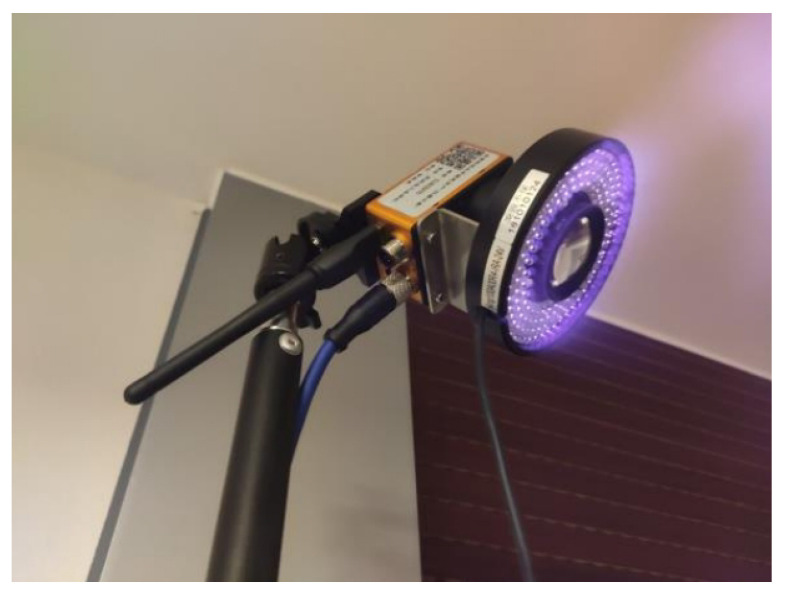
Image of the camera used in the experiments.

A sample image of the actual system is shown in Figure 7. We used three CatchBest CZE130MGEHD cameras, equipped with three AZURE-0420MM lenses (the focal length is 4 mm, and the field of view is 77.32${}^{\circ }$). Each camera has an infrared light-emitting diode (LED) light source and an infrared pass filter (850 nm wavelength), as shown in Figure 8. The image resolution is 640 px × 480 px. The ground computer used to process the data was a laptop with a 2.70 GHz AMD Ryzen 7 4800H Core processor and 16G RAM.

The experimental design is now introduced. We first needed to complete the multi-camera calibration for the UMCLS and verify the quality of calibration results through the root mean square (RMS) of the reprojection error of each camera. Secondly, after multi-camera calibration, a reconstruction experiment was performed by using some reflective spheres. The accuracy of the 3D localization function is illustrated by calculating the errors between the reconstructed distances and the actual distances. Finally, we used the system to complete the fixed-height flight task of an FWAV.

**Figure 9:**
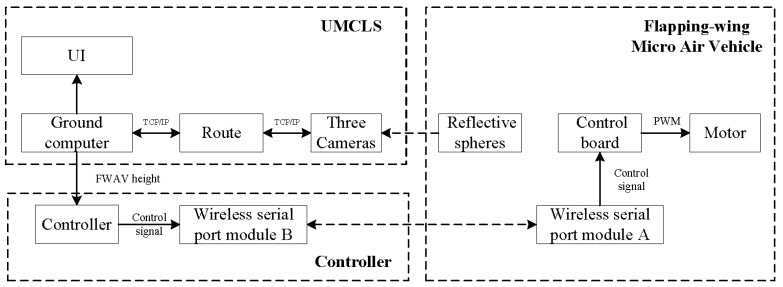
System diagram of fixed-height flight task for the flapping-wing micro air vehicle.

**Figure 10:**
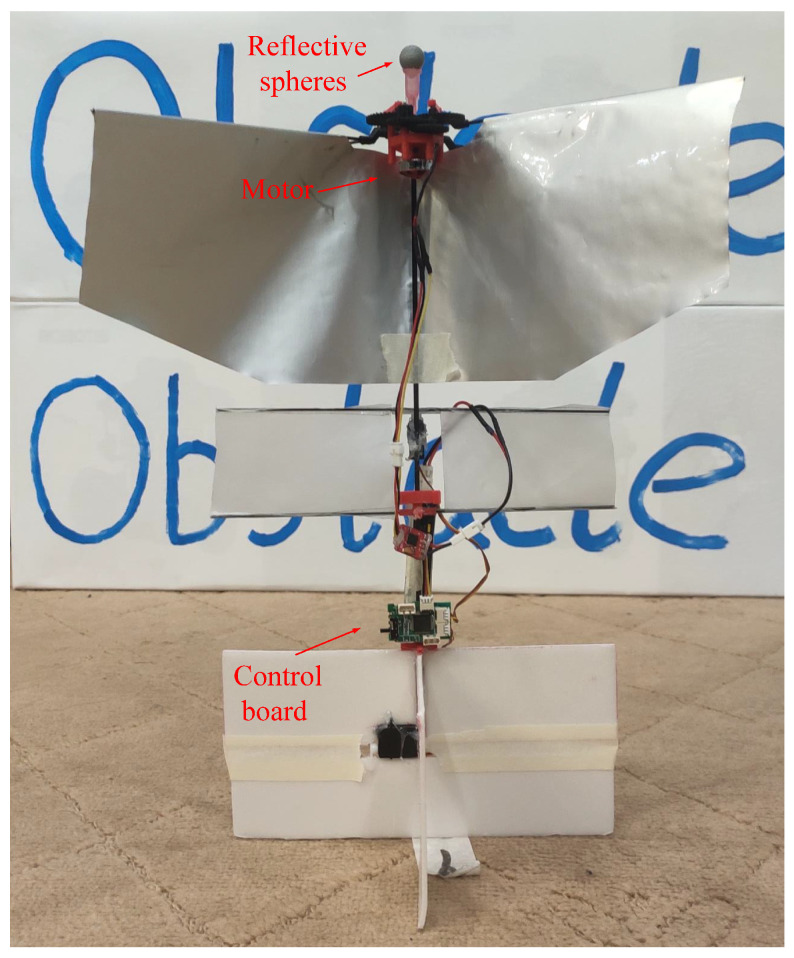
Flapping-wing micro air vehicle.

The system diagram of the fixed-height flight mission is shown in Figure 9. We fixed a reflective ball on the FWAV. The 3D coordinates of the reflective sphere were reconstructed through the system. The required control command was then calculated by a PID controller. The control signal was sent to the FWAV through the wireless serial port module. After receiving the control signal, the FWAV completes the control of the motor speed and, then, accomplishes the closed-loop control of the fixed-height task. Figure 10 shows the FWAV used in this paper. There are three main components: reflective sphere, motor, and control board.

### 3.2. Experimental Result

#### 3.2.1. Calibration Experiment

We directly verified the proposed camera calibration method through real experiments. As shown in Figure 6, three infrared reflective balls A,B,C were placed on the 1D calibration wand. The distances between them satisfy the following.

(22) $$\begin{array}{cc} L_{1} & =∥A-B∥=130\;\mathrm{mm}\\ L_{2} & =∥B-C∥=260\;\mathrm{mm}\\ L & =∥A-C∥=390\;\mathrm{mm} \end{array}$$

Firstly, we performed subsequent calibration experiments on the stereo cameras. By changing the frame rates of the cameras, we compared the performance of the proposed method in this paper with that of the method mentioned in [9]. Each experiment was performed three times. The results are shown in Table 1, Table 2 and Table 3.

It can be found from Table 1 that the proposed method can deal with the unsynchronized problem caused by the different frame rates of the cameras, while the method in [9] cannot. Through the results of Table 2 and Table 3, the proposed method can not only solve the unsynchronized problem caused by different frame rates, but also that caused by different communication delays.

Then, we adopted the above three cameras to perform multi-camera calibration by using the proposed method. We kept the frame rates of Cam0 and Cam1 as identical, and by changing their frame rates, we conducted two sets of experiments. The calibration results are shown in Table 4 and Table 5.

Since the method in [9] cannot deal with the unsynchronized problem, the calibration results of the three cameras were not compared for the different methods. It can be found from Table 4 and Table 5 that, as the discrepancy of the camera frame rates decreases, the reprojection errors of these cameras generally decrease as well, which is in line with the actual situation. Note that the reprojection errors of Cam0 are relatively large in Table 4. This is probably because the delay of network transmission has a considerable influence on the multi-camera calibration results.

#### 3.2.2. Localization Experiment

After multi-camera calibration, we evaluated the accuracy of the 3D localization by reconstructing the 3D coordinates of the three reflective spheres. The specific implementation was to calculate and compare the reconstructed distances and the actual distances. We evaluated the reconstruction accuracy for the multi-camera calibration results under different circumstances, and the results are shown in Table 6. It was found that, although the frame rates of multiple cameras varied, the reconstruction errors did not obviously change. Note that the maximum reconstruction error was less than 20 mm, which can meet the requirements of many experiments.

#### 3.2.3. Fixed-Height Experiment

**Figure 11:**
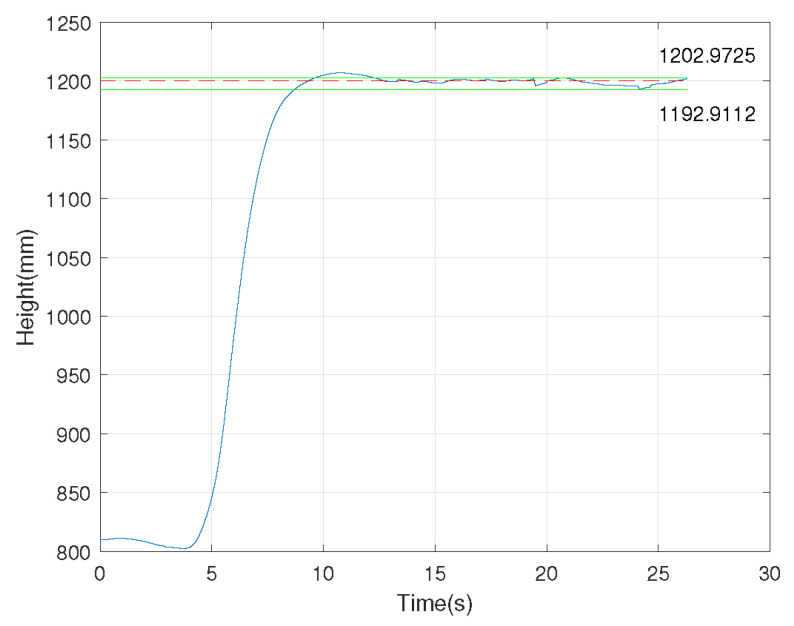
Fixed-height experiment of 1200 mm.

**Figure 12:**
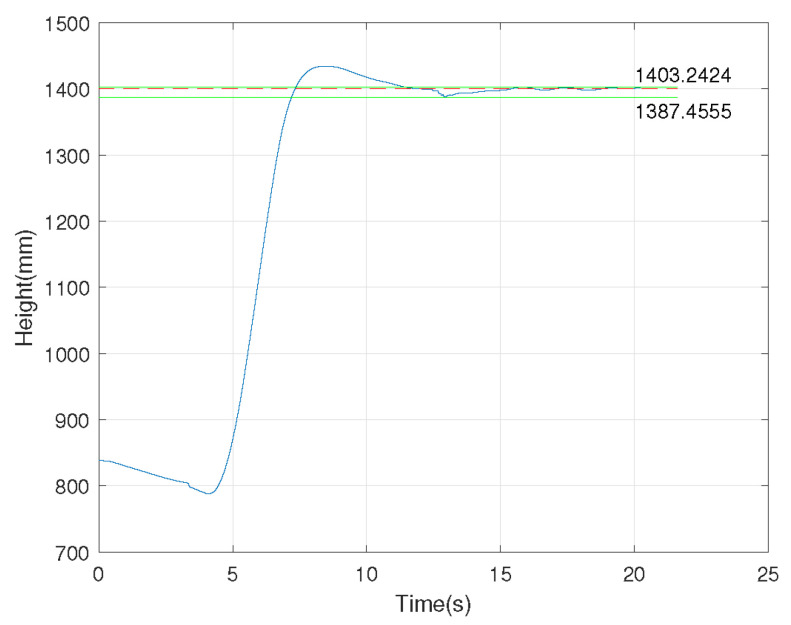
Fixed-height experiment of 1400 mm.

We investigated the performance of the UMCLS established in this paper through a fixed-height experiment of the FWAV shown in Figure 10. The fixed reflective sphere on the FWAV can be reconstructed through the UMCLS to obtain its position information in real-time. The control quantity was calculated by the PI control method, and the control signal was sent to the FWAV control board to realize the closed-loop control. Note that three cameras were used in the experiment, of which two cameras (with frame rates of 70 Hz and 80 Hz) communicated with the ground computer through a network cable and the remaining camera (with a frame rate of 110 Hz) communicated with the ground computer through WiFi. We completed two fixed-height experiments of 1200 mm and 1400 mm, and the results are shown in Figure 11 and Figure 12, respectively. It can be concluded that the established UMCLS performed well for the fixed-height experiments, and both of the control errors were guaranteed to be within 20 mm.

Note that only three cameras were adopted in this paper and the coverage area of 3D localization was quite limited. In the future, more cameras can be used so that the coverage area is increased and the FWAV could perform more tasks.

## 4. Conclusions

In this paper, a wand-based calibration method was proposed to calibrate the internal and external parameters of unsynchronized multiple cameras. Compared with the traditional calibration methods, the superiority of the proposed calibration method is that it can solve the unsynchronized problem caused by different camera frame rates and communication delay. Combined with the extended Kalman filter, a 3D localization algorithm for unsynchronized multiple cameras was proposed. In addition, an actual UMCLS was established with three smart cameras, and the practicality of the localization system was verified through fixed-height control experiments of an FWAV. This paper is helpful for researchers who want to build a UMCLS for 3D localization by using inexpensive and off-the-shelf cameras.

## Figures and Tables

**Table 1:** RMS reprojection errors of Cam0 (40 Hz) and Cam1 (70 Hz).

Experiment Number	Proposed (Pixel, Pixel)	[9] (Pixel, Pixel)
1	(1.308,1.731)	(>100, >100)
2	(0.811,1.048)	(>100, >100)
3	(1.285,1.560)	(>100, >100)

**Table 2:** RMS reprojection errors of Cam0 (60 Hz) and Cam2 (110 Hz).

Experiment Number	Proposed (Pixel, Pixel)	[9] (Pixel, Pixel)
1	(0.836,1.098)	(>100, >100)
2	(3.404,3.340)	(>100, >100)
3	(3.636,4.346)	(>100, >100)

**Table 3:** RMS reprojection errors of Cam1 (80 Hz) and Cam2 (110 Hz).

Experiment Number	Proposed (Pixel, Pixel)	[9] (Pixel, Pixel)
1	(1.891,2.032)	(>100, >100)
2	(0.684,0.751)	(>100, >100)
3	(0.821,0.794)	(>100, >100)

**Table 4:** RMS reprojection errors of Cam0 (60 Hz), Cam1 (60 Hz), and Cam2 (110 Hz).

Experiment Number	Cam0 (Pixel)	Cam1 (Pixel)	Cam2 (Pixel)
1	7.780	2.427	2.743
2	6.382	4.734	4.445
3	5.329	2.405	2.650

**Table 5:** RMS reprojection errors of Cam0 (90 Hz), Cam1 (90 Hz), and Cam2 (110 Hz).

Experiment Number	Cam0 (Pixel)	Cam1 (Pixel)	Cam2 (Pixel)
1	3.850	3.385	2.936
2	5.314	1.066	1.143
3	4.247	2.975	2.539

**Table 6:** Reconstruction errors of three calibration results.

Error (mm)	60-60-110 HZ	75-75-110 HZ	90-90-110 HZ
1	7.9496	12.1173	8.2264
2	1.3425	18.5684	1.6776
3	4.3363	5.6703	16.1708

## Data Availability

The data are contained within the article.
